# Genetic and epigenetic alterations of steroidogenic factor-1 in ovarian tumors

**DOI:** 10.3892/ijo.2012.1758

**Published:** 2012-12-28

**Authors:** SARAH MILLER, NOBEL BHASIN, HEATHER URREGO, KRZYSZTOF MOROZ, BRIAN G. ROWAN, MEERA S. RAMAYYA, NICK M. MAKRIDAKIS

**Affiliations:** 1 The Ohio Department of Health, Columbus, OH 43215;; 2 Department of Epidemiology and Tulane Cancer Center, Tulane University; New Orleans, LA 70112;; 3 Departments of Pathology, Tulane University School of Medicine, New Orleans, LA 70112;; 4 Structural and Cellular Biology and Tulane Cancer Center, Tulane University School of Medicine, New Orleans, LA 70112;; 5 Department of Pediatrics, John A. Burns School of Medicine, University of Hawaii, Honolulu, HI 96813, USA

**Keywords:** somatic, mutation, ovarian, carcinogenesis, steroidogenesis

## Abstract

Steroidogenic factor-1 (SF-1), the product of the NR5A1 gene, is an essential transcription factor that is known to regulate steroidogenesis in ovarian epithelia, including the synthesis of progesterone, a suppressor of ovarian cancer. Expression of the SF-1 protein, a potential ovarian tumor suppressor, has been demonstrated in normal OSE cells, but is lost in most ovarian tumors and ovarian tumor cell lines. We examined loss of heterozygosity (LOH) and promoter methylation as potential mechanisms that may explain the loss of SF-1 protein in ovarian tumor tissues. Genotyping of three NR5A1 SNPs in matched tumor/normal tissues identified LOH in 16/36 (44%) of the ovarian tumors successfully analyzed, and somatic mutations (gain of allele) in 10% of the tumors. Furthermore, a methylation-sensitive restriction enzyme method was used to demonstrate statistically significant (p<0.0001) increase in the frequency of NR5A1 gene methylation in ovarian tumors (36/46; 78%) versus normal ovaries (1/11; 9%). These data suggest that the SF-1 encoding gene exhibits frequent genetic (LOH/base substitution) and epigenetic (methylation) somatic alterations in ovarian tumors. These data also present novel molecular mechanisms that may explain the loss of SF-1 protein in ovarian tumors, and its potential role in ovarian carcinogenesis.

## Introduction

Ovarian cancer is the most frequent cause of death from gynecologic neoplasm in the Western World, mainly due to lack of early detection and understanding of its etiology (1). Most ovarian malignancies have epithelial origin and are often derived from ovarian surface epithelial (OSE) cells (2). Thus, understanding the molecular mechanisms that control OSE cell proliferation and differentiation may lead to the design of novel targeted therapies. Normal human OSE cells are capable of steroidogenesis (3–5) and in epithelial ovarian cancer (EOC), intratumoral steroid biosynthesis is closely linked with carcinogenesis (2,5,6).

Several studies support a role for SF-1 as a suppressor of ovarian cancer: i) ectopic expression of SF-1 inhibits rat ovarian epithelial cell proliferation, causing cell cycle arrest and promoting apoptosis (7); ii) the tumor suppressor Rb1 synergizes with steroid receptor co-activator-2 (SRC-2) to enhance the activity of SF-1 as well as nuclear receptors ERα and ERβ (8); Rb1 may thus promote the transcription of target genes linked to cell differentiation; iii) SF-1 promotes differentiation of human and rat granulosa cells associated with the developing oocytes (9).

It is well established that OSE cell proliferation and ovarian steroidogenesis are closely linked (2,5,6). Specifically, both cell culture and epidemiologic data support a protective role for progesterone against ovarian cancer (10,11). In addition to upregulating steroidogenic enzymes p450scc and 3β-HSD II, SF 1 stimulates expression of the human *StAR* gene (12). The expression and functional integrity of the StAR protein and enzymes p450scc and 3β-HSD II are particularly important for progesterone biosynthesis (13).

We have previously shown that while human SF-1 and StAR are expressed in normal OSE cells, ovarian cancer cell lines SKOV-3, OVCar3 and BG1 do not show SF-1 or StAR expression (14). We then utilized immunohistochemistry to demonstrate that the vast majority of the human ovarian tumor tissues examined do not express SF-1 protein (unpublished data). In addition, real-time PCR studies on epithelial ovarian cancers suggest that StAR mediated progesterone biosynthesis may inhibit OSE tumor cell proliferation (15). Collectively these studies support the hypothesis that loss of SF-1 protein may contribute to carcinogenesis in ovarian epithelial cells, in part, through decreased progesterone biosynthesis.

It is noteworthy that the human *NR5A1* gene has been mapped to chromosome 9 at position 9q33 (16), a region that shows genetic alterations (LOH, microsatellite instability, and amplification) in more than half of ovarian tumors (17). Particularly significant is the observation that nearly all of the tumors that show genetic alterations at 9q include the subchromosomal region 9q32–34, suggesting that a candidate tumor suppressor gene may reside in this region (17). The location of human SF-1 in the region of 9q33 supports our hypothesis that SF-1 is a candidate tumor suppressor gene in the ovary and that abolished or aberrant SF-1 expression in OSE cells may promote tumor growth. We thus decided to examine the degree of LOH in ovarian tumors, specifically at the NR5A1 locus and report LOH in 44% of the tumors.

Methylation controls the time and cell-type specific NR5A1 gene expression in the endocrine system (18). Thus we examined the methylation status of the NR5A1 gene promoter in ovarian tumors, and report significantly higher prevalence of NR5A1 gene methylation in ovarian tumors compared to normal (i.e. non-tumor) ovaries. These data suggest that LOH and methylation may contribute to the loss of SF-1 protein in ovarian tumors, which in turn may result in ovarian carcinogenesis.

## Materials and methods

### 

#### Samples

Following approval of a research protocol by the Institutional Review Board Committees of Tulane University and Louisiana State University in New Orleans, 66 archival formalin-fixed paraffin-embedded (FFPE) tissue blocks of ovarian tissue were obtained from the Departments of Pathology at the Tulane University Health Sciences Center and from the Interim LSU Hospital. Sixteen samples were representative of normal ovaries from women who had undergone gynecological surgeries for non-ovarian related causes. The rest of the 50 samples consisted of 3 benign ovarian tumors, 7 tumors of low malignancy potential and 40 cases of ovarian carcinoma. Each case of ovarian tumor was matched with corresponding benign tissue control. All FFPE tissue blocks were sectioned and stained with hematoxylin and eosin for histological assessment.

### DNA extraction

#### FFPE tissue blocks

Tissue (1.5–2 mg) was excised from each normal and tumor FFPE tissue block using a sterile scalpel and placed in an autoclaved 1.5 ml centrifuge tube. Samples were deparafinized with 1 ml of xylene followed by vortexing at top speed for 2 min. The tissue was then centrifuged at 10,000 x g for 3 min using Microcentrifuge 16 from Beckman Coulter Inc. (Brea, CA, USA) and the supernatant was pipetted out. To remove any residual xylene and facilitate pelleting, 1 ml 100% ethanol was added to the tissue sample, followed by spinning at 10,000 × g. The supernatant was decanted and tissue pellets were allowed to air-dry at room temperature. Subsequently, pellets were subjected to protease digestion by 100 *μ*l/ml proteinase K in Digestion Buffer [10 mM Tris-HCl (pH 8.0), 1 mM ethylenediaminetetraacetic acid (EDTA)] at 52°C for 16 h. Following the digestion, DNA was isolated using the Qiagen (Valencia, CA, USA) DNeasy for FFPE kit, following the manufacturer’s recommended protocol.

#### Normal ovarian tissue samples

Ovarian surface epithelial cells from normal ovarian FFPE tissue samples were dissected using the PALM^®^ Robot Microbeam laser microdissection system (PALM GmbH, Bernried, Germany) at the Louisiana State University Morphology and Imaging core facility. DNA was then extracted from the epithelial cells using the proteinase K DNA extraction Solution and incubation at 65°C for 16 h, as suggested by the manufacturer (Arcturus^®^, Applied Biosystems, Life Technologies Corporation, Carlsbad, CA, USA).

#### Genotyping

Both tumor and normal DNA samples were genotyped for SNPs: rs2279605, rs10120967, rs7851737 using Applied Biosystems TaqMan probes, with IQ power mix (Bio-Rad, Hercules, CA, USA) or Amplitaq Gold, 25 mM MgCl_2_ and 10X PCR Gold buffer from Applied Biosystems and dNTPs from VWR International (Radnor, PA, USA). Applied Biosystems Taq Man probes are labeled with Fam and Vic dyes. A total of 20 *μ*l PCR reactions were set up in a 96-well plate which was covered with Microseal ‘B’ film from Bio-Rad. Bio-Rad Thermal cycler IQ5 was used to run the real-time PCR and Image Quant 5 software from Bio-Rad used for plate read document and analysis of the real-time data post PCR. All genotyping assays were done in triplicates and when the three independent assays yielded ambiguous results, were repeated again.

#### Digestion of genomic DNA for methylation study

DNA (0.5 *μ*g) from each sample (tumor and normal from the same patient) was digested using 5 units of Afe1 enzyme (New England Bioscience, Ipswich, MA, USA) in a total reaction volume of 50 *μ*l. The digestion was performed in 1X NEBuffer (New England Bioscience); 1X NE buffer contains: 20 mM Tris-acetate, 50 mM potassium acetate, 10 mM magnesium acetate, 1 mM dithiothreitol (pH 7.9 at 25°C). The samples were incubated with the enzyme for 1 h at 37°C to allow digestion of DNA, following which Afe1 was inactivated by incubating the samples at 65°C for 20 min. Alternatively, digestion was performed overnight.

#### PCR amplification for methylation study

All samples were simultaneously PCR-amplified for the promoter region of the β-actin gene and *NR5A1* gene in 200 *μ*l tubes. For each 50 *μ*l reaction, 2 *μ*l of DNA was used and reagent concentrations were optimized at: 5 mM for MgCl_2_ from Applied Biosystems, 2 mM for each dNTP from VWR; 5 mM for primers (β-actin, forward primer: 5′-TGC AAA GAA CAC GGC TAA GTG TGC-3′, β-actin, reverse primer: 5′TCT AAG ACA GTG TTG TGG GTG TAG GTs-3′, *NR5A1* gene, forward primer: 5′-AAC ACC AAC AAA GAA GGC GAG AGG-3′, *NR5A1* gene, reverse primer: 5′-TCA CTT ACG AAG CGG AAG CAGC-3′) from IDT DNA (Coralville, IA, USA) in 10X PCR buffer II [final concentration: 50 mM potassium chloride and 10 mM Tris-HCl (pH 8.3 at room temperature) from Applied Biosystems] along with 1.25 units AmpliTaq^®^ DNA polymerase per 50 *μ*l of reaction. PCR amplification was performed on a PTC-100™ programmable thermocycler from MJ Research Inc. (Quebec, Canada) allowing initial denaturation at 95°C for 20 min followed by 40 cycles of 95°C for 1 min, 62°C for 1 min, 72°C for 1 min and completing the terminal extension with 10 min at 72°C.

#### Gel electrophoresis

A total of 8 *μ*l of PCR product was added to 2 *μ*l of loading dye (2% xylene cyanol, 40% glycerol in DDi H_2_O) from Boston Bioproducts (Ashland, MA, USA). For sizing 1 kb plus DNA ladder (Invitrogen, Life Technologies Corporation, Carlsbad, CA, USA) was loaded on a 2% agarose gel containing 1X Tris-acetate-EDTA buffer (40 mM Tris acetate and 1 mM EDTA) and 5 *μ*g of ethidium bromide for staining. The gel was run on a horizontal system for Gel electrophoresis from Bethesda Research Laboratories Inc. (Gaithersburg, MD, USA) at 100 V for 60 min. Following the gel electrophoresis the amplified fragments were visualized on Molecular Imager^®^ Gel Doc™ using Image Lab™ software from Bio-Rad.

#### Methylation analysis

Electrophoretic images were analyzed for relative (NR5A1/β-actin) band intensity using Image Quant 5.1 software from GE Healthcare (Piscataway, NJ, USA). Relative intensities were categorized in quartiles as follows: −, 1st; + 2nd; ++ 3rd; +++ 4th quartile. All experiments were done at least twice and the relative intensities were averaged.

#### Clinical application

A retrospective chart review was performed gathering clinical data on the patients for whom we had malignant ovarian tissue. Characteristics examined were: age, race/ethnicity, date of diagnosis, years survived since diagnosis, stage of ovarian cancer, histologic grade, date of debulking surgery and treatment with chemotherapy or radiation.

#### Statistics

For most statistical calculations, two-tailed p-values were obtained using Fisher’s exact test. The log-rank test was used for calculating p-values for potential differences in survival.

## Results

### 

#### Samples

The clinical characteristics of the ovarian tumor samples examined are shown on Table I.

#### LOH at the NR5A1 locus in ovarian tumors

In the current study, we considered two molecular hypotheses of SF-1 protein loss in ovarian tumors: LOH and increased methylation. To probe for the prevalence of LOH at the *NR5A1* locus, we genotyped matched ovarian tumor and normal FFPE tissue DNA samples from the same ovarian cancer patient, for three *NR5A1* gene SNPs: rs2279605, rs10120967 and rs7851737. SNPs were selected based on the following criteria: i) high (>30%) frequency of heterozygosity in the racial/ethnic groups present in our study population (based on available data at dbSNP: http://www.ncbi.nlm.nih.gov/projects/SNP/); ii) availability of a preoptimized Taqman SNP Genotyping Assay for each SNP. Genotyping was performed by Taqman SNP Genotyping Assays using real-time PCR. Assays were performed in triplicates and repeated again, if the genotyping results were ambiguous. All three genotyped SNPs were in Hardy-Weinberg equilibrium in normal samples (data not shown).

**Figure 1.**
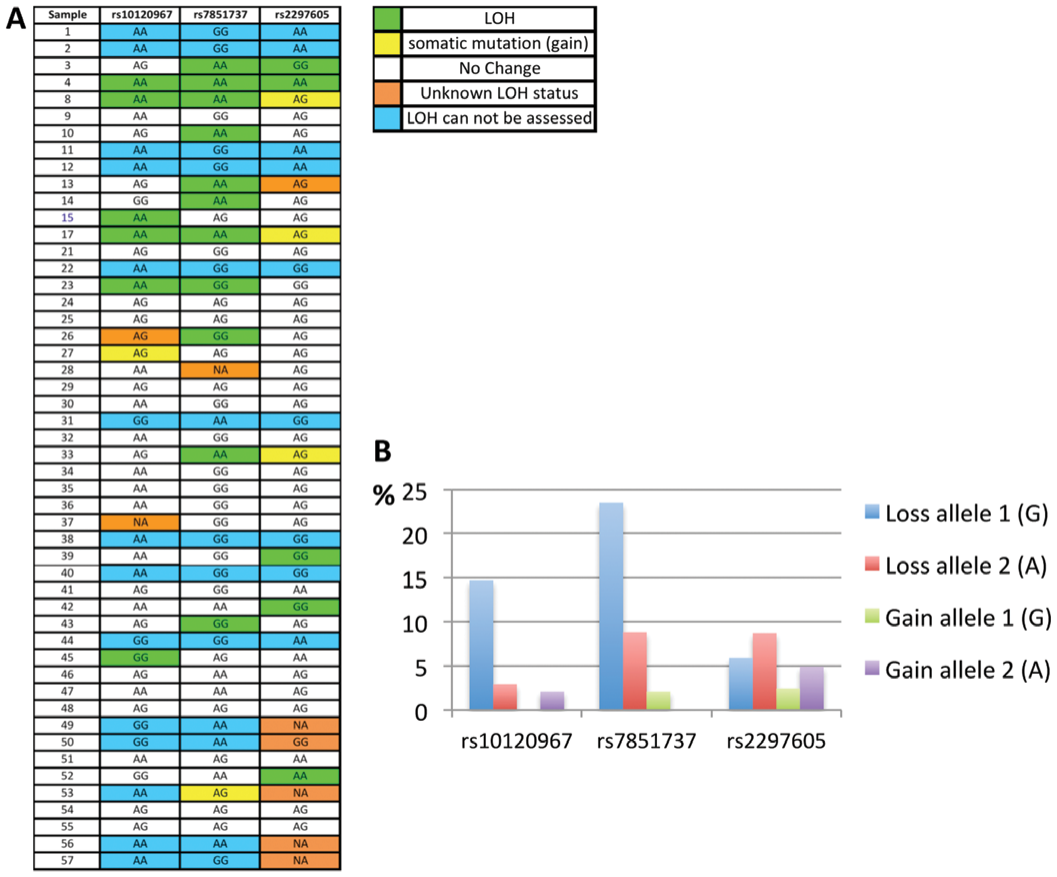
Genotyping reveals somatic NR5A1 gene alterations in ovarian tumors. (A) Genotype distribution by sample. Somatic mutations were identified by genotyping matched ovarian tumors and normal tissue from the same individuals by Taqman SNP genotyping asssays for the indicated SNPs. Only tumor genotypes are shown. (B) Genotyping distribution by SNP. Key: LOH, loss of heterozygosity (e.g. AG>AA); gain, gain of heterozygosity (e.g. GG>AG). Unknown, unclear genotype, for either tumor or normal sample.

**Figure 2.**
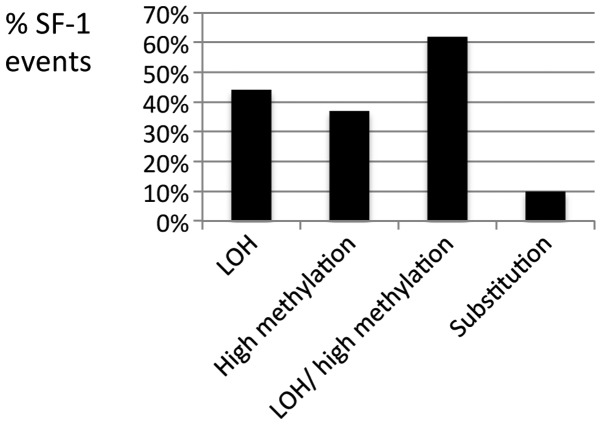
NR5A1 gene alterations in ovarian tumors. Key: High methylation = 3rd and 4th quartile of methylation levels (see Materials and methods). Substitution, a somatic mutation other than LOH (eg. AA>AG). LOH/High methylation, LOH or high methylation.

The genotyping results for the ovarian tumors and the LOH data for each sample, are shown in Fig. 1A. These data show that out of the 36 ovarian tumor tissues that were heterozygous for at least one of the three *NR5A1* gene SNPs, 16 (44%) had LOH (Figs. 1A and 2). The majority of the ovarian tumors had a single LOH event at the *NR5A1* locus (out of maximum three possible), but 5 tumors (14%) showed multiple LOH events (Fig. 1A). Also, each SNP showed LOH in multiple tumors, with rs7851737 showing most losses (Fig. 1B). Thus, LOH occurs frequently at the *NR5A1* locus in ovarian tumors.

With regards to the type of observed loss, LOH events at rs2297605 were equally distributed between the two alleles, while the other two SNPs showed bias in the LOH events towards one of the two alleles (Fig. 1B). The significance of this finding is unclear, since these are non-coding SNPs. Interestingly, the genotyping results (Fig. 1A) also uncovered somatic mutations other than LOH in the tumors, manifested as allele gains; base substitutions turning a homozygous genotype (normal DNA) into a heterozygote genotype in the tumor, hence called ‘gain of allele’; Fig. 1B. These somatic *NR5A1* substitutions were present in 10% of ovarian tumors (Fig. 2). Thus the genotyping data show frequent genetic (LOH/substitution) events at the *NR5A1* locus in ovarian tumors.

#### Methylation of the NR5A1 gene in ovarian tumors

A methylation-sensitive restriction enzyme (Afe1) method (e.g. 19) was used to quantify site-specific methylation at −30 bp (compared to translation start) of the *NR5A1* gene promoter in ovarian tumor tissue from patients with ovarian cancer and in matched normal tissue from the same patients (when available).

Afe1 cleaves genomic DNA at 5′-AGC/GCT-3′, but cleavage is blocked by methylation (http://www.neb.com/nebecomm/products/productr0652.asp). Since the Afe1 enzyme cleaves the un-methylated CpG’s, only methylated CpG’s can be amplified and quantified following gel electrophoresis. Complete DNA digestion was confirmed by prolonged (overnight) Afe1 digestion, which yielded similar results (data not shown). To control for differences in DNA level and/or quality between tumor samples, we also amplified β-actin as an internal control. The primers used for the β-actin gene were selected to amplify a region that lacks an Afe1 cleavage site. Therefore, β-actin is amplified regardless of methylation status, and relative band intensity (*NR5A1*/β-actin) was used as a function of *NR5A1* gene methylation (see Materials and methods for details). This analysis indicated that 36 out of 46 (78%) ovarian tumors had appreciable *NR5A1* methylation (2nd, 3rd and 4th quartile of methylation levels), and 17/46 (37%) had high levels of NR5A1 methylation (3rd and 4th quartile of methylation levels; Table II). Thus the *NR5A1* gene is methylated in most ovarian tumors. Furthermore, we detected both a high level of *NR5A1* gene methylation and LOH in 21% of the ovarian tumors that we analyzed (Fig. 1 and Table II). The cumulative data also demonstrate that 62% of the ovarian tumors have LOH, high level of methylation, or both (Fig. 2) at the *NR5A1* locus.

As indicated by Table I, most ovarian tumors are diagnosed at an advanced stage, reducing the ability to obtain normal ovarian tissue from most patients. In the absence of an adequate number of matched normal ovaries available for study, 16 non-tumor ovaries were analyzed (from unrelated individuals) to evaluate the relative methylation of the promoter region of the *NR5A1* gene in normal ovarian tissue, with the same methylation-sensitive restriction enzyme method used above. Since human ovarian tumors have epithelial origin (2,20), we obtained OSE cells from these ovarian tissues (by laser-capture microdissection) and analyzed them following DNA extraction. β-actin was again amplified from each sample as an internal control. These data show that only one out of the 11 (9%) normal ovaries that were successfully evaluated (i.e. that had β-actin amplification) showed appreciable *NR5A1* methylation (Table III). This difference between the prevalence of *NR5A1* methylation in tumor versus normal ovaries is statistically significant (p<0.0001). Thus, ovarian tumor tissues display significantly more frequent *NR5A1* gene methylation than normal ovarian epithelial tissues.

#### Clinical correlation

Retrospective analysis of the clinical data suggest that presenting stage and histologic grade of ovarian tumors are not significantly affected by the presence of somatic *NR5A1* gene alterations (Table I and data not shown). Furthermore, Kaplan-Meier survival curves were similar for both ovarian tumors with and without *NR5A1* gene alteration (LOH/methylation; data not shown). Likewise, the presence of *NR5A1* gene alteration did not correlate with race/ethnicity or treatment, such as radiation and chemotherapy (Table I and data not shown).

## Discussion

A common feature of many tumor suppressor genes is their inactivation in cancer tissue through LOH and other somatic mutations. In ovarian tumors, LOH and somatic mutations have been documented for tumor suppressors such as *TP53*, *BRCA1*, *BRCA2* and *PTEN*(21). The data presented herein support such a role for human SF-1, and may provide a molecular mechanism to partially explain the loss of SF-1 protein reported in both ovarian tumors and ovarian cancer cell lines. Specifically, the data demonstrate that most ovarian tumors contain genetic and/or epigenetic alterations at the *NR5A1* locus, significantly more frequently compared to normal ovaries. These somatic alterations include LOH, base substitution, and methylation of the *NR5A1* gene promoter. The absence of correlation between the presence of somatic *NR5A1* gene alteration and disease treatment (radiation/chemotherapy) suggests that these somatic events are not the result of cancer treatment. These data suggest the need for scanning the *NR5A1* gene for somatic mutations in larger datasets, with diverse racial/ethnic groups, and perhaps in other types of tumor tissues controlled by SF-1.

Given the prevalence of somatic events at the *NR5A1* locus, we attempted to examine the contribution of these molecular events on clinical endpoints, such as disease progression and survival. Interestingly, genetic and epigenetic *NR5A1* alterations do not correlate with markers of tumor progression (grade/stage) or survival. This finding suggests that somatic *NR5A1* alterations may be early events in ovarian carcinogenesis. Analysis of the early stage/grade tumors in our dataset suggests a similar prevalence of somatic *NR5A1* alterations in advanced and non-advanced tumors (data not shown). However, this interpretation is tempered by the existence of low numbers of non-advanced tumors in our dataset (Table I). Examination of larger numbers of non-advanced and/or benign tumors for somatic *NR5A1* alterations, may help confirming this concept.

The finding of somatic *NR5A1* gene mutations (gain of allele substitutions) in 10% of the ovarian tumors (Fig. 2) is striking, given the fact that we interrogated only three base pairs of the *NR5A1* gene in these assays (the three SNP positions). This fact together with the finding of LOH at this locus in 44% of the ovarian tumors (Fig. 2), strongly suggest a high somatic mutation frequency of the *NR5A1* gene in ovarian cancer. Thus, the *NR5A1* gene should be screened for somatic mutations by a more comprehensive method (such as sequencing) in both advanced and benign ovarian tumors, especially tumors that show LOH. This analysis should include the *NR5A1* gene promoter, since SF-1 protein expression is lost in both human ovarian tumors and tumor cell lines. Identification of a somatic mutation and/or methylation together with LOH in the same tumor, may explain the loss of SF-1 protein reported in ovarian tumor tissue. To that effect, the detection of both a high level of *NR5A1* gene methylation and LOH in 21% of the ovarian tumors that we analyzed (Fig. 1 and Table II), may partially explain this loss.

LOH can be caused by two different mechanisms in tumor cells: i) deletion (loss of allele/gene) or ii) base substitution (which includes gene conversion). Although 14% of the tumors had multiple LOH events (Fig. 1) suggesting a deletion at the *NR5A1* locus, the majority of LOH events involved only one (out of three possible) SNPs at the *NR5A1* locus (Fig. 1), suggesting no extensive *NR5A1* deletion, at least around the three interrogated SNPs. However, even a microdeletion involving only the genomic area around a single *NR5A1* SNP can affect SF-1 protein expression. Also, gene conversion involves recombination (22), which may cause deletions, rearrangements and other functionally important (for SF-1 expression) genetic events upstream or downstream from the interrogated SNPs (undetected by our assay). Furthermore, both molecular heterogeneity within the same tumor and contamination with normal cells can result in underestimation of the extent of LOH, or confinement of the observed LOH in a smaller genetic region. In addition, the high somatic mutation frequency observed at the *NR5A1* locus may have functional effects. Therefore, the frequently observed genetic (LOH/substitution) events at the *NR5A1* locus may significantly contribute to the reported loss of SF-1 protein in ovarian tumor tissue.

A limitation of the methylation-sensitive restriction enzyme method utilized here is that the use of PCR, which exponentially amplifies the target DNA, makes the method less quantitative. For this reason, we included β-actin as an internal control, and also focused our methylation analysis on quartiles of methylation rather than absolute levels. The significant difference observed in the frequency of appreciable methylation (2nd quartile or higher) between tumor and normal ovarian tissue (Tables II and III) suggests that *NR5A1* methylation is much more prevalent in tumors. The exact degree of methylation, and the subsequent reduction of SF-1 protein, is hard to estimate from these data. However, the fact that the 37% of the ovarian tumors that show high methylation (++ or higher methylation level; Fig. 2) display >57% of the band intensity of β-actin (Table II), suggests that a significant proportion of the NR5A1 gene is methylated in these ovarian tumors in vivo. Thus methylation may be a major molecular mechanism leading to the reported loss of SF-1 protein in ovarian tumors.

Interestingly, hypomethylation and subsequent transcriptional activation of SF-1 has been reported in endometriosis, an estrogen dependent disease (23). In contrast, hypermethylation leading to silencing of gene expression has been reported in ovarian tumors for multiple key tumor suppressor genes including *BRCA1*, *BRCA2*, *WT1*, *APC*, *CDKN2A* and *MLH1*(24,25).

In conclusion, we report frequent somatic alterations of the NR5A1 locus in ovarian tumors, including LOH, base substitution, and methylation of the NR5A1 gene promoter. These molecular abnormalities may partially explain the loss of SF-1 protein, and contribute to the model of SF-1 as an ovarian tumor suppressor. The existence of both genetic and epigenetic NR5A1 gene abnormalities in ovarian tumors further suggest that SF-1 is a common and important target in ovarian carcinogenesis.

## Figures and Tables

**Table I.** Clinical characteristics of ovarian tumors studied.

Age	Race	Date of Dx	Study no.	Years survived since Dx	Stage	Histological grade	Surgery	Chemo	Radiation
53	Black	6/13/07	T23	Unknown	3C	3	6/13/07	Yes	No
62	White	6/19/07	T24	<1 year	3C	3	6/19/07	No	No
32	White	4/19/07	T25	<1 year	4	3	8/23/07	No	No
54	Black	12/12/07	T26	Living	0	Borderline	12/12/07	No	No
31	White	1/15/08	T27	Living	0	Borderline	1/15/08	No	No
26	Black	2/18/08	T28	Living	3B	1	2/18/08	No	No
45	Hispanic	4/28/08	T29	Living	0	2	4/29/08	No	No
64	White	5/8/08	T30	Living	0	Borderline	5/18/08	No	No
60	White	8/13/08	T31	Living	3C	3	8/13/08	Yes	No
47	Black	10/8/08	T32	Living	0	Borderline	10/8/08	No	No
55	White	10/9/08	T33	1 year	3C	2	10/9/08	No	No
72	Unknown	10/27/08	T34	Living	3C	3	10/27/08	Yes	No
57	White	12/7/08	T35	<1 year	3C	Borderline	12/7/08	Unknown	Unknown
74	Hispanic	12/11/08	T36	Unknown	3C	3	12/11/08	Yes	No
50	Black	1/29/09	T37	<1 year	4	2	1/29/09	No	No
49	Black	5/11/09	T38	Living	0	Borderline	5/11/09	No	No
56	Black	9/28/09	T39	Living	3A	2	9/28/09	Yes	Yes
70	Unknown	10/1/09	T40	Living	3C	3	10/1/09	Yes	No
46	Black	3/2/07	T21	Unknown	3C	3	3/3/07	Yes	No
41	Black	6/12/07	T22	1 year	3C	3	6/11/07	Yes	No
73	White	4/24/97	T10	1 month	3B	3	11/24/97	No	No
41	Black	7/21/98	T8	2 years	4	3	7/21/98	Yes	No
69	White	4/5/99	T13	6 years	4	3	4/5/99	Yes	No
63	Black	3/5/00	T9	2 years	3C	2	3/5/00	Yes	No
47	White	5/31/00	T14	Living	3B	3	5/31/00	Yes	No
26	White	5/21/99	T15	3 years	3C	3	5/21/99	Yes	Yes
61	Black	3/5/02	T49	<1 year	4B	2	3/5/02	Yes	No
36	White	5/29/02	T50	Living	0	Borderline	5/29/02	No	No
60	White	6/28/02	T11	Unknown	3C	2	6/28/02	Unknown	Unknown
83	White	8/9/02	T51	<1 year	3C	3	8/9/02	No	Yes
77	Hispanic	3/28/03	T52	<1 year	4B	3	6/13/03	Yes	No
67	Black	9/24/03	T53	3 years	3C	3	9/26/03	Yes	No
43	White	6/18/01	T54	2 years	NA	3^a^ colon primary	6/18/01	Yes	No
47	White	12/19/03	T17	Living	3A	3	12/19/03	Yes	No
68	Black	2/16/04	T55	<1 year	4	3	2/16/04	No	No
75	White	6/20/03	T1	2 years	4	3	6/20/03	Yes	No
35	Hispanic	5/25/05	T41	<1 year	3C	2	5/25/05	Yes	No
76	White	7/20/06	T42	2 years	3C	3	10/24/06	Yes	No
45	White	10/21/02	T12	4 years	2A		10/21/02	No	No
60	NA	2/10/02	T4	<1 year	4	3	3/5/02	Yes	No
51	White	3/13/07	T3	Living	3C	3	3/13/07	Yes	No
70	Indian	3/16/07	T2	Unknown	4	3	3/16/07	Unknown	Unknown
65	White	6/8/07	T44	<1 year	4	3	6/12/07	No	No
68	Black	8/17/07	T45	Living	4	3^a^ endometrial cancer	8/17/07	No	Yes
64	White	6/23/08	T56	Living	3C	3	7/9/08	Yes	No
49	White	6/25/09	T46	Living	2B	Unknown	6/25/09	No	No
66	Hispanic	7/16/09	T47	Living	3C	3	7/16/09	Yes	No
51	White	8/8/09	T48	Living	1A	1	8/5/09	No	No
30	White	8/5/09	T57	Living	1A	Borderline	8/5/09	No	No

Key: Borderline/stage 0 tumors, indicate atypical changes in the ovary that have low malignancy potential. Dx, diagnosis.

**Table II.** NR5A1 gene methylation in ovarian tumors.

Samples	Relative intensity	Methylation level
T1	0.24	−
T2	0.81	++
T3	1.14	+++
T4	0.78	++
T8	0.35	+
T9	0.72	++
T10	0.02	−
T11	0.34	+
T12	0.40	+
T13	0.77	++
T14	0.48	+
T15	0.54	+
T17	0.40	+
T21	0.45	+
T22	0.62	++
T23	0.58	++
T24	0.56	+
T25	0.68	++
T26	0.37	+
T27	0.87	+++
T28	0.55	+
T29	0.78	++
T30	0.83	++
T31	0.58	++
T32	0.09	−
T33	0.57	+
T34	0.32	+
T35	0.17	−
T36	0.53	+
T37	0.43	+
T38	0.54	+
T39	0.57	++
T40	0.42	+
T41	NA	NA
T42	0.75	++
T43	NA	NA
T44	0.14	−
T45	0.00	−
T46	1.14	+++
T47	NA	NA
T48	NA	NA
T49	0.10	−
T50	0.11	−
T51	0.79	++
T52	0.53	+
T53	1.07	+++
T54	0.00	−
T55	0.00	−
T56	0.54	+
T57	0.30	+

Methylation was quantified by the methylation-sensitive restriction enzyme method. Relative intensity, NR5A1/β-actin band intensity. Methylation level is indicated by quartiles of relative intensity:

−, 1st;

+, 2nd;

++, 3rd; and

+++, 4th quartile.

**Table III.** NR5A1 gene methylation in normal ovaries.

Samples	Relative intensity	Methylation level
N41	0	−
**N61**	1.5	+++
N44	0.1	−
N34	NA	NA
N45	0.16	−
**N60**	0	−
N42	0	−
**N59**	0	−
N52	0	−
**N29**	NA	NA
N51	0	−
**N38**	0	−
N54	NA	NA
N53	0.21	−
N57	NA	NA
**N27**	NA	NA

Bold indicates samples with available tumor. The numbers for normal (N) tissue are scrambled, and do not correspond to the tumor (T) tissue numbers. For key, see Table II.

## Abbreviations:

SF-1: steroidogenic factor-1
StAR: steroidogenic acute regulatory protein
OSE: ovarian surface epithelial
ERα: estrogen receptor α
ERβ: estrogen receptor β
EOC: epithelial ovarian cancer
Rb1: retinoblastoma 1
SNP: single nucleotide polymorphism
